# The association between death anxiety and nutrition awareness in elderly hospitalized patients: a dual-perspective analysis from person-centered and variable-centered approaches based on future time perspective

**DOI:** 10.3389/fnut.2025.1741292

**Published:** 2026-01-23

**Authors:** Lijun Li, Dan Wu, Qin He, Jianao Chen

**Affiliations:** 1 Department of Rehabilitation, Chongqing Hospital of The First Affiliated Hospital of Guangzhou University of Chinese Medicine (Chongqing Beibei Hospital of Traditional Chinese Medicine), Chongqing, China; 2 Geriatrics Department, Hangzhou TCM Hospital Affiliated to Zhejiang Chinese Medical University, Hangzhou, China; 3 Department of Science and Education, Hangzhou TCM Hospital Affiliated to Zhejiang Chinese Medical University, Hangzhou, China

**Keywords:** death anxiety, elderly hospitalized patients, future time perspective, nutrition awareness, person-centered and variable-centered

## Abstract

**Background:**

With the acceleration of global population aging, psychological health issues among elderly hospitalized patients have become increasingly prominent. Death anxiety, as a key psychological experience of existential threat, is associated with psychological adaptation challenges in older adults and may be related to health behavior intentions. Nutrition awareness is a critical factor in promoting rehabilitation and healthy aging; however, the associations between death anxiety and nutrition awareness, as well as potential psychological pathways, remain understudied.

**Objective:**

This study aims to examine the associations among death anxiety, future time perspective, and nutrition awareness, while identifying group heterogeneity through latent profile analysis.

**Methods:**

This study employed a cross-sectional design, recruiting elderly hospitalized patients (*N* = 557) from three tertiary Grade-A hospitals in Hangzhou and Chongqing, China. Measurements were conducted using the Death Anxiety Scale, Future Time Perspective Scale, and Nutrition Awareness Scale. Data analyses included descriptive statistics, correlation analysis, structural equation modeling, and latent profile analysis.

**Results:**

Death anxiety was significantly and positively associated with both future time perspective and nutrition awareness. Future time perspective demonstrated a significant indirect effect in the relationship between death anxiety and nutrition awareness, suggesting a partial mediation pattern. Latent profile analysis identified three distinct subgroups: “low death anxiety–low future time perspective,” “moderate death anxiety–moderate future time perspective,” and “high death anxiety–high future time perspective,” with the latter group demonstrating the highest levels of nutrition awareness.

**Conclusion:**

Under the moderating influence of future time perspective, death anxiety can be transformed into a positive psychological driver for health-promoting behaviors. Enhancing future orientation and sense of life meaning in elderly patients can convert anxious emotions into motivation for health management, providing a scientific basis for individualized psychological-nutritional interventions.

## 1 Introduction

With the acceleration of global population aging, elderly hospitalized patients have emerged as an increasingly prominent group within healthcare systems (1, 2). However, due to physiological functional decline, increased chronic diseases, shifts in social roles, and heightened loneliness, elderly patients often experience significant psychological stress responses (3, 4). Among these, death anxiety is widely recognized as one of the most representative and far-reaching psychological experiences, serving as a key factor influencing elderly health (5). Death anxiety refers to an individual’s fear and unease regarding the process of death, its unknown consequences, and the termination of life (6). Previous studies have shown that death anxiety is closely associated with anxiety, depression, and social withdrawal in elderly hospitalized patients, and may also be related to quality of life and rehabilitation processes through its associations with health behavior choices and the construction of life meaning (7, 8). However, existing research has predominantly focused on the negative correlates of death anxiety, with limited analyses examining whether individuals facing death threats might demonstrate stronger health intentions and self-care awareness. In hospital settings, death anxiety may be associated with older adults’ reevaluation of their lifestyles (9), and nutrition awareness in elderly hospitalized patients may represent an important psychological-behavioral factor related to health recovery and aging processes.

Nutrition awareness refers to an individual’s cognition and emphasis on nutritional needs, dietary choices, and the benefits of healthy eating (10, 11). According to terror management theory (TMT), when individuals become aware of their own finitude, they alleviate anxiety by strengthening their sense of self-worth and life meaning, which could be associated with stronger health responsibility awareness (12, 13). In elderly hospitalized patients, death anxiety, as a signal of existential threat, may be associated with reevaluation of future health states and willingness for behavioral adjustments (14). Specifically, death anxiety may be related to heightened awareness of life’s finitude, potentially leading patients to reassess life priorities and view nutrition as a means to maintain quality of life (15). Thus, this study seeks to address: (1) whether death anxiety in elderly hospitalized patients is significantly associated with nutrition awareness; and (2) whether future time perspective demonstrates an indirect effect in this relationship, suggesting a potential mediating pathway.

Future time perspective refers to an individual’s subjective perception of future time and its psychological orientation in life planning (16). A positive future time perspective encourages individuals to evaluate life benefits from a long-term viewpoint, thereby emphasizing health investment behaviors such as nutrition management and disease prevention (17); conversely, a limited future time perspective often leads to short-sighted avoidance tendencies and behavioral negativity (18). Existing research indicates that future time perspective plays a significant mediating role between health threats and health behaviors (19), influencing how individuals interpret death threats and the meaning of self-care. For elderly hospitalized patients, when they possess high future time openness, death anxiety may no longer be perceived as a destructive psychological experience but as an important opportunity for life adjustment and health reconstruction. In contrast, if the future is perceived as limited or ambiguous, death anxiety is prone to transform into despair and abandonment.

Based on socioemotional selectivity theory (SST) and TMT (12, 20), future time perspective may serve as a potential mediating variable in the relationship between death anxiety and nutrition awareness. When elderly hospitalized patients confront death threats, death anxiety may function as a psychological signal of existential distress, triggering a reevaluation of future time (21). Among individuals with a high future time perspective—that is, those perceiving the future as open and full of possibilities—the perception of death will shift from fear to a motivational source, thereby activating intrinsic needs to maintain health and extend quality of life. In this process, future time perspective regulates individuals’ cognitive and emotional responses, transforming death anxiety from negative avoidance into a positive driver for health behaviors (5). Specifically, future orientation may be associated with patients’ emphasis on long-term health benefits, including nutrition management and self-care awareness (22); additionally, positive future perceptions may be related to reduce destructive effects of anxiety, potentially supporting the reconstruction of life meaning and psychological security (23). Thus, future time perspective not only integrates the logical connections between death anxiety and health behaviors at the cognitive level but also facilitates the psychological transformation from existential threats to motivational activation at the emotional and meaning-construction levels (24), thereby forming an internal mechanism in elderly hospitalized patients whereby death anxiety is converted into enhanced nutrition awareness and strengthened health responsibility.

### 1.1 Theoretical gaps and novel contributions

Despite the well-established foundations of TMT and SST, several critical theoretical gaps remain when these frameworks are applied to health behaviors among elderly hospitalized patients. First, TMT has predominantly conceptualized responses to mortality salience as defensive mechanisms aimed at anxiety reduction, including worldview defense, self-esteem striving, and symbolic immortality pursuits (25, 26). However, TMT has been largely silent on whether and how mortality awareness might be associated with concrete, adaptive health behaviors rather than purely psychological defenses. The theory’s emphasis on proximal and distal defenses against death-related cognition has inadvertently positioned death anxiety primarily as a threat to be managed rather than a potential resource to be channeled (27, 28). This represents what we term the “defensive bias” in TMT—an overemphasis on anxiety reduction at the expense of understanding anxiety utilization. Our study addresses this gap by examining whether death anxiety, rather than merely triggering defensive responses, may be associated with proactive health engagement in the form of enhanced nutrition awareness.

Second, SST posits that perceived limitations in future time lead to prioritization of emotionally meaningful goals over knowledge-acquisition goals (29). While this framework has been extensively applied to social relationship preferences and emotional regulation (30, 31), its application to health behaviors has produced inconsistent findings. Some studies suggest that limited future time perspective should reduce investment in long-term health behaviors (17), while others find that elderly individuals maintain or increase health-promoting behaviors despite perceiving limited time (32). This inconsistency represents what we term the “health behavior paradox” in SST—the theory does not adequately explain why some older adults with limited future time perspective nonetheless demonstrate strong health investment. Our study addresses this gap by examining how future time perspective may function as a cognitive framework that differentially relates death awareness to health behavioral intentions.

Third, and most critically, neither TMT nor SST adequately addresses the intersection of death anxiety and future time perspective in predicting health outcomes. TMT treats mortality salience as a threat that activates defenses, while SST treats time perspective as a motivational framework that shapes goal priorities (33). However, these theories have operated largely in parallel, with limited theoretical integration regarding how existential threat and temporal cognition might jointly relate to health behaviors. Our study proposes that future time perspective may serve as a cognitive-interpretive framework that is associated with whether death anxiety co-occurs with health-promoting or health-neglecting behavioral patterns—a proposition that neither theory alone can fully address.

To address these gaps, we introduce the concept of “constructive anxiety” as a novel theoretical extension. We define constructive anxiety as a pattern wherein existential distress co-occurs with adaptive behavioral orientations, characterized by the channeling of mortality awareness toward life-affirming health investments rather than defensive withdrawal or fatalistic disengagement. This concept extends TMT beyond its traditional focus on anxiety reduction to encompass anxiety utilization, and it refines SST by specifying conditions under which future time perspective may be associated with the adaptive correlates of existential awareness. The identification of a “high death anxiety–high future time perspective” subgroup with elevated nutrition awareness provides preliminary empirical support for this construct, though we acknowledge that cross-sectional data cannot establish whether this pattern reflects a causal transformation of anxiety into constructive outcomes.

### 1.2 The study

The variable-centered approach focuses on average relationships between variables (34), such as regression analysis examining the overall effect of death anxiety on nutrition awareness; the person-centered approach, through latent profile analysis, identifies heterogeneous subgroups (35), revealing how different latent subgroups of death anxiety and future time perspective influence nutrition awareness. Existing research has largely been limited to the variable-centered perspective, leading to the neglect of individual differences. To address the shortcomings of prior studies, this research innovatively adopts a dual perspective from person-centered and variable-centered approaches, constructing a mediation model of “death anxiety → future time perspective → nutrition awareness” to explore the complex dynamics of health psychology in elderly hospitalized patients. Unlike previous studies that relied solely on macro-variable analyses, this study emphasizes the importance of individual differences, balancing the stability of group models with the heterogeneity of individual psychological traits, thereby providing a new theoretical framework for understanding the positive potential of death anxiety from a psychological adaptation perspective. At the nursing practice level, the results of this study can offer a scientific basis for developing individualized psychological interventions and nutritional guidance strategies, promoting healthy aging by optimizing psychological resources and social support systems for hospitalized elderly patients.

## 2 Methods

### 2.1 Participants

This study strictly adhered to international ethical standards, including the Declaration of Helsinki and its amendments. All procedures were reviewed and approved by the Academic Ethics Committee of Hangzhou TCM Hospital (No.: 2025KLL074) and Chongqing Hospital of The First Affiliated Hospital of Guangzhou University of Chinese Medicine (Chongqing Beibei Hospital of Traditional Chinese Medicine) (No.: BBQZYYEC-2025-0801). Prior to the study, detailed informed consent forms were provided to all potential participants, explaining the study’s purpose, procedures, potential risks, and benefits. All participants received comprehensive study explanations before commencing and signed informed consent forms. Researchers ensured participant privacy throughout data collection and processing, with all data anonymized and encoded solely for academic research purposes. Participants had the right to withdraw from the study at any stage without affecting their medical treatment.

This study employed a cross-sectional design to examine the associations among death anxiety, future time perspective, and nutrition awareness in elderly hospitalized patients at a single time point. This design allows for the simultaneous collection of data on multiple variables to reveal inter-variable correlations but cannot establish causal relationships. The research framework was based on a mixed-methods approach, integrating variable-centered and person-centered perspectives: the variable-centered approach used structural equation modeling to test overall pathways, while the person-centered approach employed latent profile analysis to identify heterogeneous subgroups.

Participants were recruited from April to September 2025, with data collected in the geriatric wards of three tertiary Grade-A hospitals in Hangzhou and Chongqing, China. Recruitment strategies involved convenience sampling combined with systematic screening. Hospital nursing staff reviewed daily admission lists to initially identify individuals meeting age criteria, after which researchers approached potential participants, provided study overviews, and assessed interest. To enhance participation rates, multi-channel promotion was used, including ward posters, verbal explanations, and discussions involving family members, emphasizing the non-invasive nature of the study and its potential benefits. To minimize information bias, measurements were conducted when patients were alert and in stable physical condition, with the survey process averaging 5–8 min.

To ensure sample representativeness and study validity, strict inclusion and exclusion criteria were established. Inclusion criteria were as follows: (1) age ≥ 65 years; (2) currently undergoing inpatient treatment with hospitalization duration ≥ 7 days; (3) diagnosed with chronic diseases (e.g., cardiovascular diseases, diabetes, or respiratory diseases); (4) able to comprehend and complete the questionnaire (Mini-Mental State Examination (MMSE) score ≥ 24) (36); (5) voluntary participation with written informed consent.

Exclusion criteria included: (1) severe cognitive impairment or dementia (MMSE score < 24) to avoid compromising the accuracy of self-reported data; (2) acute psychiatric disorders or current intensive care status; (3) hospitalization duration < 7 days to exclude transient effects of short-term hospitalization on psychological variables; (4) inability to independently complete the questionnaire or requiring proxy responses; (5) history of severe malnutrition or eating disorders to control potential confounding factors; (6) refusal to participate or withdrawal of consent. These criteria were implemented through initial screening and clinical assessments, reviewed by two independent researchers to ensure consistency.

Although strict inclusion and exclusion criteria were applied to ensure the internal validity of the measurements, the sampling strategy in this study followed a convenience-based approach within three tertiary Grade-A hospitals in Hangzhou and Chongqing. This approach was selected due to practical constraints in accessing elderly inpatient populations and the need to recruit participants who met the cognitive, medical, and procedural requirements of the study. While tertiary Grade-A hospitals in China typically serve a broad spectrum of elderly patients and provide relatively standardized medical services, the sample cannot be assumed to fully represent elderly hospitalized populations in other regions, hospital tiers, or socioeconomic contexts. Therefore, the findings should be interpreted with caution regarding external generalizability.

To determine the minimum sample size, comprehensive power analyses were conducted considering the distinct requirements of each primary analytical method employed in this study. For structural equation modeling (SEM), sample size determination followed established guidelines. According to Kline (37), a minimum of 200 participants is recommended for SEM analyses, with a preferred ratio of at least 20:1 (cases to free parameters). Our mediation model, which included death anxiety as the independent variable, future time perspective as the mediator, and nutrition awareness as the dependent variable along with three covariates, contained approximately 9 free parameters, thus requiring a minimum of 180 participants. Additionally, Wolf, Harrington (38) demonstrated through Monte Carlo simulations that for simple mediation models with standardized path coefficients of medium magnitude (*β* ≈ 0.39), a sample size of 148 is sufficient to achieve 80% statistical power. To ensure robust parameter estimation and model fit assessment, we targeted a sample exceeding 500 participants. For latent profile analysis (LPA), sample size considerations followed recommendations by Nylund-Gibson and Choi (39). Simulation studies indicate that samples of 300–500 participants are generally adequate for identifying 2–4 latent classes with acceptable classification accuracy (entropy > 0.80). Furthermore, each identified class should contain a minimum of 5% of the total sample (approximately 25–50 cases) to ensure stable parameter estimation and meaningful clinical interpretation (40). Given our expectation of identifying 3–4 latent profiles based on theoretical considerations and prior research, a target sample of at least 400 participants was deemed necessary. Additionally, *a priori* power analysis using G*Power 3.1 software was conducted for subsidiary regression analyses. Assuming a medium effect size (f^2^ = 0.15), significance level (*α*) of 0.05 (two-tailed), statistical power (1-*β*) of 0.95, and 9 predictor variables including demographic covariates, the minimum required sample was 107 participants.

Integrating these considerations, we established a target sample size of at least 500 elderly hospitalized patients to ensure adequate statistical power across all primary analytical approaches while accounting for potential data attrition. Our final effective sample of 557 participants (efficiency rate: 94.56%) substantially exceeded the minimum requirements for SEM (≥180), LPA (≥400), and regression analyses (≥107), thereby ensuring robust and reliable statistical inferences. Post-hoc sensitivity analysis confirmed that this sample size achieved statistical power exceeding 0.95 for detecting the observed effect sizes in both mediation and latent profile analyses.

Accordingly, a total of 589 questionnaires were distributed in this study. Among them, 9 patients refused to sign informed consent forms, 14 had hospitalization durations less than 1 week, 6 had MMSE scores below 24, and 3 had questionnaires with strong response consistency. A total of 32 participants were excluded, resulting in an effective sample size of 557 and an efficiency rate of 94.56%. There were 342 male elderly hospitalized patients (61.40%) and 215 female elderly hospitalized patients (38.60%). Educational backgrounds were primarily concentrated among patients with primary school education or below (*N* = 274, 49.20%), with 285 patients residing in urban areas (51.20%) and 272 in rural areas (48.80%). Marital status was predominantly married (*N* = 320, 57.50%), and 204 patients had been hospitalized for 31–60 days (36.60%). The mean age of elderly hospitalized patients was 75.268 (SD = 6.974). Detailed information is presented in Table 1.

**Table 1:** Summary of demographic information for elderly hospitalized patients.

Variables	Items	Number	Proportion
Gender	Male	342	61.40%
	Female	215	38.60%
Educational background	Primary school and below	274	49.20%
	Junior high school - Senior high school	128	23.00%
	Bachelor’s degree or above	155	27.80%
Place of residence	City	285	51.20%
	Rural	272	48.80%
Marital status	Married	320	57.50%
	Unmarried	48	8.60%
	Divorced	70	12.60%
	Widowed	119	21.40%
Length of stay	7–14 days	67	12.00%
	15–30 days	145	26.00%
	31–60 days	204	36.60%
	61 days or more	141	25.30%
Age	75.268 ± 6.974		

### 2.2 Clarifying the construct of death anxiety

Although death anxiety is commonly conceptualized as an individual’s fear and apprehension toward death and dying (41), contemporary literature emphasizes that it is not a unitary construct. Instead, death anxiety encompasses several distinguishable dimensions that vary in temporal stability, awareness level, and thematic focus. First, researchers differentiate between trait death anxiety, representing relatively stable individual differences in death-related fear (42), and state death anxiety, which refers to short-term fluctuations triggered by situational cues (43). Second, based on terror management theory and mortality salience research, death anxiety can be further divided into conscious and nonconscious levels, with the latter influencing behavior through automatic defensive processes (44, 45). Third, structural analyses of death-related fears suggest further differentiation between individuals’ fear of the dying process (e.g., pain, loss of autonomy) and fear of the aftermath of death (e.g., nonexistence, consequences for loved ones) (46).

In the present study, we operationalized death anxiety using the Templer Death Anxiety Scale (47), which primarily assesses conscious, trait-like death anxiety and integrates both fear-of-dying-process and fear-of-aftermath components within its item structure. This approach aligns with prior research in clinical geriatric populations and provides stable estimates suitable for cross-sectional mediation and latent profile analyses (48). Nevertheless, we acknowledge that this operationalization does not capture the full spectrum of death anxiety, particularly nonconscious mortality salience or momentary state fluctuations. These distinctions also suggest that different subcomponents of death anxiety may have divergent associations with health behavior motivation. Therefore, future research may extend the present model by incorporating multidimensional or implicit measures of death anxiety to determine whether specific death-related fears differentially influence future time perspective and nutrition awareness.

### 2.3 Measures tools

#### 2.3.1 Death anxiety scale

Items for measuring death anxiety were derived from the Death Anxiety Scale developed by Templer (47), which consists of 15 items and is a unidimensional scale. This scale was translated into Chinese by Yang, Zhang (49) and validated for cultural adaptability and reliability among colorectal cancer patients. Additionally, it has been widely applied to middle-aged and elderly Chinese inpatients with chronic diseases (8). In this study, the scale was used to assess death anxiety in elderly hospitalized patients, employing a 5-point Likert scale ranging from 1 = strongly disagree to 5 = strongly agree. Higher scores indicate stronger death anxiety emotions in patients.

Confirmatory factor analysis (CFA) was conducted using Mplus 8.3 with maximum likelihood estimation to evaluate the unidimensional structure of the Death Anxiety Scale in the current sample. The results indicated acceptable model fit: χ^2^/df = 2.271, CFI = 0.972, TLI = 0.968, RMSEA = 0.048, GFI = 0.953, AGFI = 0.937. All 15 items demonstrated adequate standardized factor loadings ranging from 0.577 to 0.737, exceeding the recommended threshold of 0.50 (50). No item-level modifications (e.g., item deletions or correlated error terms) were applied, as the original model achieved satisfactory fit. The Cronbach’s alpha for the Death Anxiety Scale was 0.933, indicating excellent internal consistency reliability.

#### 2.3.2 Zimbardo time perspective inventory

Items for measuring future time perspective were derived from the Zimbardo Time Perspective Inventory (51). This inventory comprises 56 items across five subscales: Past-Negative, Present-Hedonistic, Future, Past-Positive, and Present-Fatalistic. The focus of this study was on the Future subscale, which reflects orientation toward achieving future goals and features planning characteristics, consisting of 13 items. Thus, this study utilized the Future subscale to assess future time perspective in elderly hospitalized patients, a method widely applied in previous research (52), and translated into Chinese by Li, Wang (53). A 5-point Likert scale was used, ranging from 1 (strongly disagree) to 5 (strongly agree). Higher scores indicate stronger future time perspective regarding survival quality in patients.

CFA was performed to verify the unidimensional structure of the Future Time Perspective subscale. The model demonstrated good fit to the data: χ^2^/df = 4.304, CFI = 0.888, TLI = 0.865, RMSEA = 0.077, GFI = 0.923, AGFI = 0.892. Standardized factor loadings for all 13 items ranged from 0.527 to 0.615, all exceeding the 0.50 criterion. The original scale structure was retained without modifications. The Cronbach’s alpha was 0.860, indicating good internal consistency reliability.

#### 2.3.3 Nutrition awareness scale

Items for measuring nutrition awareness were derived from the Nutrition Awareness Scale by van Dillen, Hiddink (54), which consists of 17 items. In this study, the scale was used to assess nutrition awareness in elderly hospitalized patients, employing a 5-point Likert scale (1 = strongly disagree, 5 = strongly agree). Higher scores indicate higher nutrition awareness in elderly hospitalized patients. In this study, the Cronbach’s alpha for the nutrition awareness items was 0.886, indicating good internal consistency. Confirmatory factor analysis confirmed that the scale exhibited good structural validity in the Chinese inpatient population.

CFA was conducted to evaluate the structural validity of the Nutrition Awareness Scale in the Chinese elderly hospitalized patient population. The unidimensional model demonstrated acceptable fit: χ^2^/df = 3.314, CFI = 0.897, TLI = 0.882, RMSEA = 0.065, GFI = 0.919, AGFI =. All 17 items exhibited standardized factor loadings between 0.526 and 0.613, meeting the recommended threshold. No post-hoc modifications were made to the model structure. The Cronbach’s alpha was 0.886, indicating good internal consistency reliability.

### 2.4 Statistical analysis

All statistical analyses were performed using SPSS software (version 27.0), PROCESS macro (version 4.1), and Mplus software (version 8.3). First, descriptive statistics were conducted, including means, standard deviations, frequencies, and percentages, to summarize sample characteristics and variable distributions. Skewness (< |3|) and kurtosis (< |8|) within these ranges were considered indicative of normal distribution (37).

To examine inter-variable relationships, Pearson correlation coefficients were first calculated to assess bivariate correlations among death anxiety, nutrition awareness, and future time perspective. Subsequently, Hayes’ PROCESS macro (Model 4) was employed to analyze the mediating role of future time perspective (55). Specifically, this model tested the pathway through which death anxiety (independent variable) influences nutrition awareness (dependent variable) via future time perspective (mediator). Bootstrapping resampling (5,000 iterations) was used to estimate the 95% confidence intervals (CI) for indirect effects, assessing the significance of mediation. If the CI did not include zero, the mediation effect was significant. Covariates, including gender, marital status, and hospitalization duration, were included in the model to control for potential confounders.

Furthermore, to explore the heterogeneity of death anxiety and future time perspective, latent profile analysis was conducted using Mplus software. LPA was based on continuous indicators of these two variables to identify potential subgroup profiles. Model fit indices included the Akaike Information Criterion (AIC), Bayesian Information Criterion (BIC), sample-adjusted BIC (aBIC), entropy, and Lo–Mendell–Rubin likelihood ratio test (LMR-LRT). Models were incrementally increased from 1 class until the optimal model was identified (based on the lowest BIC, significant LMR-LRT, and high entropy, typically >0.80). Average scores and probabilities for each profile were used to describe subgroup characteristics, with multi-group comparisons examining differences in nutrition awareness across profiles.

## 3 Results

### 3.1 Common method bias

This study employed Harman’s single-factor test, incorporating all measurement items from the core variables into an exploratory factor analysis. The results revealed seven factors with eigenvalues greater than 1, with the first factor accounting for 27.005% of the total variance, below the critical threshold of 40%. Therefore, no common method bias was present in this study. To further mitigate potential common method bias, based on prior research, data collection incorporated measures such as ensuring confidentiality and anonymity to minimize bias (56).

### 3.2 Descriptive and correlation analyses

Descriptive statistics and correlation analyses were conducted for death anxiety, future time perspective, and nutrition awareness, as shown in Tables 2, 3. The mean score for death anxiety among elderly hospitalized patients was 3.045 (SD = 0.835), for future time perspective was 3.143 (SD = 0.692), and for nutrition awareness was 3.183 (SD = 0.671). Thus, elderly hospitalized patients exhibited relatively high levels of death anxiety, high future time perspective, and strong nutrition awareness. According to Kline (37), the skewness ranged from −0.291 to 0.050, and kurtosis ranged from −0.383 to −0.219. Therefore, the core study variables approximated a normal distribution.

**Table 2:** Descriptive statistics of core variables.

Variables	M	SD	Skewness	Kurtosis
Death anxiety	3.045	0.835	0.050	−0.335
Future time perspective	3.143	0.692	−0.221	−0.383
Nutrition awareness	3.183	0.671	−0.291	−0.219

M represents the mean value; SD stands for standard deviation.

**Table 3:** Correlation analysis of core variables.

Variables	1	2	3
Death anxiety	1		
Future time perspective	0.574***	1	
Nutrition awareness	0.382***	0.438***	1

***p < 0.001.

Based on the correlation analysis, death anxiety showed a significant strong positive correlation with future time perspective (*r* = 0.574, *p* < 0.001), indicating that death anxiety positively promoted long-term future planning and investment among elderly hospitalized patients; death anxiety exhibited a significant moderate positive correlation with nutrition awareness (*r* = 0.382, *p* < 0.001), suggesting that elderly hospitalized patients with high death anxiety regarded healthy eating as an active means to counteract death and maintain life value; future time perspective demonstrated a significant moderate positive correlation with nutrition awareness (*r* = 0.438, *p* < 0.001), implying that elderly hospitalized patients with strong future time perspective placed greater emphasis on future outcomes and focused on their nutrition and health, as shown in Table 3.

### 3.3 Mediation analysis

To enhance the accuracy of the study, one-way ANOVA was performed on demographic information and study variables. The results indicated that gender (*F*(1,555) = 63.776, *p* < 0.001), marital status (*F*(3,553) = 42.789, *p* < 0.001), and hospitalization duration (F(3,553) = 23.655, *p* < 0.001) significantly influenced patients’ nutrition awareness. Collinearity diagnostics showed variance inflation factors (VIF) < 5, indicating no severe multicollinearity (57).

Subsequently, with death anxiety as the independent variable, future time perspective as the mediator, nutrition awareness as the dependent variable, and controlling for gender, marital status, and hospitalization duration, PROCESS Model 4 was used to analyze the mediating role of future time perspective (Bootstrap: 5000). The results showed that death anxiety significantly and positively predicted future time perspective (*β* = 0.341, *p* < 0.001, 95% CI = [0.283, 0.399]); death anxiety significantly and positively predicted nutrition awareness (*β* = 0.072, *p* = 0.043, 95% CI = [0.002, 0.142]); and future time perspective significantly and positively predicted nutrition awareness (*β* = 0.243, *p* < 0.001, 95% CI = [0.152, 0.334]). Specific regression analyses are presented in Table 4.

**Table 4:** Regression models for the mediation analysis of future time perspective.

Regression equation		Overall fit index			Significance of regression coefficient				
Outcome variables	Predictive variables	R	R^2^	F	*β*	*t*	LLCI	ULCL	VIF
Future time perspective	Death anxiety	0.676	0.457	115.477***	0.341	11.557***	0.283	0.399	1.286
Nutrition awareness	Death anxiety	0.568	0.322	52.207***	0.072	2.027*	0.002	0.142	1.601
	Future time perspective				0.243	5.252***	0.152	0.334	1.844

R represents the complex correlation coefficient and R^2^ represents the proportion of variance explained by the model. β values are standardized regression coefficients, and their significance was assessed by t-test. The LLCI and ULCI are the lower and upper limits of the 95% confidence intervals, respectively. The VIF (variance inflation factor) is used to diagnose multicollinearity, and all values are below 5.

**Figure 1:**
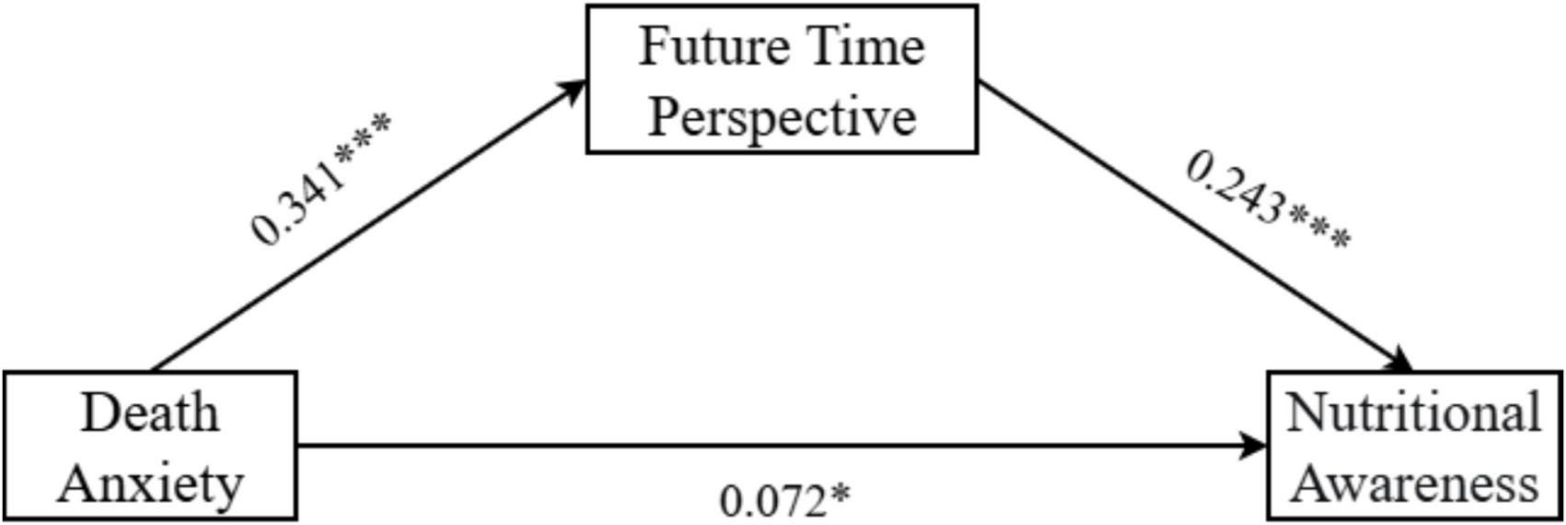
Mediating effect path coefficient of future time perspective, ***p < 0.001.

Mediation effect test for future time perspective, as shown in Table 5 and Figure 1. The total effect of death anxiety on nutrition awareness was 0.155, of which 53.55% was explained by the mediating role of future time perspective. Further analysis revealed a significant direct effect of death anxiety on nutrition awareness (*β* = 0.072, SE = 0.036, 95% CI = [0.002, 0.142]), accounting for 46.45% of the total effect. The pathway of death anxiety → future time perspective → nutrition awareness exhibited a significant partial mediating effect (*β* = 0.083, SE = 0.023, 95% CI = [0.040, 0.131]).

**Table 5:** Decomposition of the mediating effects of future time perspective.

Effect decomposition	β	SE	LLCI	ULCI	Effect proportion
Total effect	0.155	0.033	0.091	0.219	100%
Direct effect	0.072	0.036	0.002	0.142	46.45%
Indirect effect	0.083	0.023	0.040	0.131	53.55%

### 3.4 Latent profile analysis of death anxiety and future time perspective

Mplus 8.3 software was used to construct latent profile models for death anxiety and future time perspective among elderly hospitalized patients, utilizing items from the Death Anxiety Scale and Future Time Perspective Scale. Models with 1–5 classes were developed, and the optimal fit model was evaluated and selected based on model fit indices. Lower AIC, BIC, and aBIC values when comparing k-1 profile models indicated higher accuracy of the profile model. *p*-values < 0.05 for LMR and BLRT suggested that the K-class model was superior to the K-1 model. Entropy measured the precision and certainty of profile classification, with higher entropy values indicating lower classification errors, better profile differentiation, and superior classification effects (58).

As shown in Table 6, as the number of latent profile models increased, AIC, BIC, and aBIC values continuously decreased, indicating improving accuracy of the profile models. However, the Lo–Mendell–Rubin likelihood ratio test (LMR-LRT) showed that the 2-class latent profile model significantly improved over the 1-class model (*p* < 0.001), the 3-class model significantly improved over the 2-class model (*p* < 0.001), but the 4-class model was only marginally significant over the 3-class model (*p* = 0.021), and the 5-class model was not significant over the 4-class model (*p* = 0.131). The BLRT test was significant across all models (*p* < 0.001), but due to its higher sensitivity, priority was given to the conservative LMR-LRT criterion. The entropy value for the 3-class latent profile model was 0.940, indicating good classification quality (>0.80). Additionally, the minimum class proportion in the 3-class model was 0.201, ensuring sufficient sample sizes for all classes (>5%), whereas the 4-class and 5-class models exhibited smaller classes (0.038), potentially leading to instability. Therefore, this study selected the 3-class latent profile model as the optimal fit.

**Table 6:** Fit indices for latent profile models of death anxiety and future time perspective.

Profile	AIC	BIC	aBIC	Entropy	LMR (p)	BLRT (p)	Smallest proportion per class
1	48639.952	48882.015	48704.245	-	-	-	-
2	45128.726	45496.144	45226.314	0.935	<0.001	<0.001	0.645/0.355
3	43762.947	44255.720	43893.830	0.940	<0.001	<0.001	0.205/0.534/0.261
4	43243.233	43861.360	43407.411	0.953	0.021	<0.001	0.196/0.526/0.038/0.240
5	42892.650	43636.131	43090.122	0.958	0.131	<0.001	0.135/0.061/0.522/0.244/0.038

**Figure 2:**
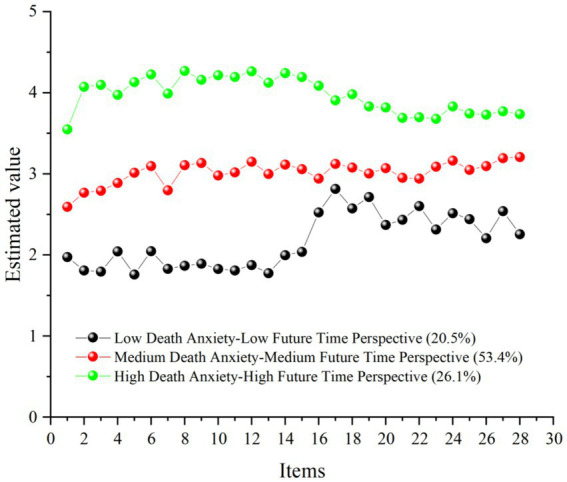
Profiles of three potential subgroups of death anxiety and future time perspective. The 1–15 measure items was titled death anxiety, and the 16–28 measure items was titled future time perspective.

Based on the analysis in Table 6, the 3-class latent profile model was selected as optimal, and profile plots for the three latent subgroups were generated using Origin 2021, as shown in Figure 2. The three latent subgroups were labeled as “low death anxiety-low future time perspective” (20.5%), which may represent a relatively calm but weakly future-oriented state; “moderate death anxiety-moderate future time perspective” (53.4%), representing moderate-level heterogeneity among the majority of patients; and “high death anxiety-high future time perspective” (26.1%), where patients, despite high anxiety, exhibited stronger positive future insights, reflecting adaptive coping mechanisms.

### 3.5 One-way ANOVA for latent subgroups and nutrition awareness

To further examine the relationship between the latent subgroups of death anxiety and future time perspective and nutrition awareness, one-way ANOVA was conducted with the three latent subgroups as the independent variable and nutrition awareness as the dependent variable. The results indicated that the nutrition awareness in the “low death anxiety-low future time perspective” group (M = 2.756, SD = 0.768) was significantly lower than in the “moderate death anxiety-moderate future time perspective” group (M = 3.152, SD = 0.407). The “high death anxiety-high future time perspective” group (M = 3.576, SD = 0.798) exhibited significantly higher nutrition awareness than the other two groups, *F*(2, 554) = 57.809, η^2^ = 0.173. Therefore, significant differences existed between the latent subgroups of death anxiety and future time perspective and nutrition awareness.

### 3.6 Exploratory analysis of potential profile characteristics

To further analyze the different configurations of demographic information in potential profile characteristics, we conducted a *post hoc* exploratory analysis, as shown in Table 7. The study indicates that based on the results of the chi-square analysis, gender, educational background, place of residence, marital status, and length of hospital stay show significant differences among different potential profiles (*p* < 0.001). There are also significant differences in the age of hospitalized patients among different potential profiles (*F* = 6.457, *p* = 0.002).

**Table 7:** Demographic and clinical characteristics across latent profiles.

Variables	Items	Profile 1	Profile 2	Profile 3	χ^2^ /F	*p*
Gender	Male	26 (23.2%)	190 (63.5%)	126 (86.3%)	107.686	<0.001
	Female	86 (76.8%)	109 (36.5%)	20 (13.7%)		
Educational background	Primary school and below	75 (67.0%)	110 (36.8%)	89 (61.0%)	45.995	<0.001
	Junior high school–Senior high school	9 (8.0%)	90 (30.1%)	29 (19.9%)		
	Bachelor’s degree or above	28 (25.0%)	99 (33.1%)	28 (19.2%)		
Place of residence	City	74 (66.1%)	135 (45.2%)	76 (52.1%)	14.335	<0.001
	Rural	38 (33.9%)	164 (54.8%)	70 (47.9%)		
Marital status	Married	100 (89.3%)	162 (54.2%)	58 (39.7%)	122.552	<0.001
	Unmarried	0 (0%)	47 (15.7%)	1 (0.7%)		
	Divorced	12 (10.7%)	31 (10.4%)	27 (18.5%)		
	Widowed	0 (0%)	59 (19.7%)	60 (41.1%)		
Length of stay	7–14 days	27 (24.1%)	33 (11.0%)	7 (4.8%)	69.232	<0.001
	15–30 days	36 (32.1%)	83 (27.8%)	26 (17.8%)		
	31–60 days	27 (24.1%)	131 (43.8%)	46 (31.5%)		
	61 days or more	22 (19.6%)	52 (17.4%)	67 (45.9%)		
Age		76.179 ± 7.145	72.298 ± 6.845	76.555 ± 6.850	6.457	0.002

Profile 1: low death anxiety–low future time perspective; Profile 2: moderate death anxiety–moderate future time perspective; Profile 3: high death anxiety–high future time perspective.

## 4 Discussion

### 4.1 Variable-centered perspective

This study systematically examined the associations among death anxiety, future time perspective, and nutrition awareness, revealing that future time perspective demonstrates a significant indirect effect in the relationship between death anxiety and nutrition awareness, consistent with a partial mediation pattern. These findings are consistent with the fundamental assumptions of terror management theory and extend its potential applicability to clinical geriatric populations. Terror management theory posits that when individuals become aware of their own finitude, they may alleviate existential anxiety by reinforcing self-worth and belongingness (59). The present study found that death anxiety in elderly hospitalized patients was positively associated with nutrition awareness, suggesting that death anxiety may not function solely as a destructive emotion but may also be related to reevaluation of life meaning and health investment. This pattern suggests that death anxiety may be associated with a “psychological activation effect” under certain conditions, characterized by its positive correlation with self-care awareness at the behavioral intention level (60). However, it is important to note that these cross-sectional findings cannot establish whether death anxiety causes changes in nutrition awareness or whether the relationship is bidirectional.

The indirect effect of future time perspective in the association between death anxiety and nutrition awareness was statistically significant. This pattern suggests that the relationship between death anxiety and nutrition awareness may be partially explained by its association with future time cognition (61). Elderly patients with higher future time openness can view health and aging issues through a more expansive temporal lens, internalizing death threats as motivational forces for life planning, thereby enhancing their self-awareness in nutritional regulation and health management. This pattern is consistent with the tenets of socioemotional selectivity theory, which posits that when future time is perceived as open rather than limited, individuals may be more inclined to pursue long-term health benefit goals (62). Notably, death anxiety demonstrated a significant direct association with nutrition awareness even when controlling for future time perspective, suggesting that the relationship between death anxiety and nutrition awareness may operate through multiple pathways—both through cognitive channels related to future time perspective and through other mechanisms that may include emotional arousal. This dual-pathway pattern suggests a complex associational structure that warrants further investigation through longitudinal designs to establish temporal precedence and potential causal mechanisms.

Drawing on cognitive-emotional interaction frameworks (63), future time perspective may be associated with both the intensity of anxiety experiences and the cognitive framing of such emotions within goal-oriented frameworks. The observed associations suggest that future time perspective may serve as a psychological factor that is related to how death anxiety is associated with health-related awareness. In other words, future time perspective may function as a cognitive bridge in the relationship between death anxiety and nutrition awareness, with higher future time perspective being associated with positive correlations between anxiety and health awareness. This pattern offers preliminary support for positive psychological interpretations of death anxiety, though longitudinal research is needed to determine whether future time perspective actually modulates or transforms the effects of death anxiety over time.

### 4.2 Person-centered perspective

#### 4.2.1 Three patterns of existential-temporal configuration

The identification of three distinct subgroups reveals that death anxiety and future time perspective do not vary independently but form characteristic configurations with different behavioral correlates. These configurations suggest that the psychological meaning of death anxiety may differ substantially depending on its co-occurrence with temporal cognitive orientations.

The “low death anxiety–low future time perspective” subgroup (20.5% of participants) demonstrated the lowest nutrition awareness. This configuration may represent what we term “existential disengagement”—a pattern characterized by reduced attention to both mortality concerns and future-oriented planning. From a theoretical standpoint, this group’s low nutrition awareness is consistent with SST predictions: limited future orientation should reduce investment in long-term health outcomes. However, the co-occurrence of low death anxiety suggests that this pattern may also reflect a form of psychological withdrawal from existential engagement rather than successful anxiety management.

The “moderate death anxiety–moderate future time perspective” subgroup (53.4% of participants) displayed intermediate nutrition awareness levels. This largest subgroup likely represents the normative experience of elderly hospitalized patients, characterized by some awareness of mortality and some future orientation, but without the pronounced patterns that might be associated with either adaptive or maladaptive extremes.

The “high death anxiety–high future time perspective” subgroup (26.1% of participants) demonstrated significantly the highest nutrition awareness. This configuration provides the clearest empirical support for the constructive anxiety concept. Despite experiencing elevated existential distress, these individuals simultaneously maintain an expansive, open orientation toward the future—and this combination is associated with the strongest health behavioral intentions. This pattern challenges the assumption that death anxiety is inherently maladaptive and suggests that its behavioral correlates depend critically on co-occurring cognitive orientations.

#### 4.2.2 Theoretical implications of the constructive anxiety profile

The “high death anxiety–high future time perspective” profile warrants particular theoretical attention because it represents a configuration that neither TMT nor SST would straightforwardly predict.

From a TMT perspective, high death anxiety typically signals failed or insufficient defensive buffering, which should be associated with psychological distress and behavioral dysfunction. Yet the individuals in this subgroup demonstrate the highest nutrition awareness, suggesting that elevated death anxiety can co-occur with adaptive behavioral orientations under specific conditions. This finding extends TMT by suggesting that the relationship between death anxiety and behavioral outcomes is not linear but may be moderated by cognitive factors such as temporal perspective.

From an SST perspective, the high future time perspective in this subgroup should indeed predict investment in future-oriented goals including health. However, SST does not explain why this future orientation co-occurs with elevated death anxiety, nor does it predict that this specific combination would be associated with particularly strong health behavioral intentions. Our findings suggest that future time perspective’s motivational effects may be amplified when combined with heightened mortality awareness—a synergistic pattern that extends SST’s explanatory scope.

We propose that the “high death anxiety–high future time perspective” profile represents a state of “existential activation”—heightened awareness of mortality combined with continued investment in a meaningful future. This configuration may reflect an adaptive integration of death awareness into life planning, wherein mortality concerns serve as motivational reminders of life’s preciousness rather than as paralyzing threats. The elevated nutrition awareness in this group may represent one manifestation of this integration: health-promoting behaviors as a means of honoring and preserving the valued future that these individuals perceive as still available to them.

#### 4.2.3 Implications for understanding individual differences

The person-centered findings underscore that average relationships between death anxiety and health behaviors obscure important individual differences. The variable-centered finding that death anxiety is positively associated with nutrition awareness represents an average effect that characterizes some individuals (particularly those with high future time perspective) much more strongly than others (particularly those with low future time perspective).

This observation has implications for both theory and practice. Theoretically, it suggests that TMT and SST must be integrated with individual-differences perspectives to adequately explain health behaviors in elderly populations. Practically, it suggests that interventions targeting death anxiety or future time perspective may have differential effects depending on individuals’ existing psychological configurations—a point we develop further in the clinical implications section.

### 4.3 Integrative analysis of variable-centered and person-centered findings

#### 4.3.1 Complementary but distinct analytical perspectives

The variable-centered mediation model and person-centered latent profile analysis offer complementary yet fundamentally different perspectives on the relationship between death anxiety, future time perspective, and nutrition awareness. Understanding how these perspectives converge and diverge is essential for a complete theoretical interpretation of our findings.

The variable-centered mediation model addresses the question: “On average, through what mechanism is death anxiety associated with nutrition awareness?” This approach assumes that the identified indirect pathway through future time perspective operates similarly across all individuals, differing only in degree based on participants’ positions along continuous variable distributions. The significant indirect effect suggests that, on average, individuals with higher death anxiety tend to report higher future time perspective, which in turn is associated with greater nutrition awareness.

The person-centered latent profile analysis addresses a fundamentally different question: “Are there qualitatively distinct subgroups of individuals characterized by different configurations of death anxiety and future time perspective, and do these subgroups differ in nutrition awareness?” This approach does not assume uniform processes across individuals but instead identifies natural clustering patterns that may reflect qualitatively different psychological states or adaptation styles.

The critical insight from integrating these approaches is that the average mediation effect identified in variable-centered analysis masks substantial heterogeneity in how death anxiety and future time perspective combine within individuals. While the mediation model suggests a general positive pathway, the LPA reveals that this pathway characterizes some individuals much more strongly than others—and that a substantial minority of participants may not follow this pathway at all.

#### 4.3.2 How LPA profiles nuance the mediation model

The three identified profiles provide crucial nuance to the mediation model in several specific ways. First, the mediation model’s indirect effect is most strongly instantiated in Profile 3 (high death anxiety–high future time perspective). This subgroup demonstrates the pattern that the mediation model describes as normative: elevated death anxiety co-occurring with elevated future time perspective, jointly associated with the highest nutrition awareness. For these individuals, the statistical mediation pathway appears to reflect a psychologically meaningful process wherein mortality awareness and temporal cognition combine synergistically to enhance health behavioral intentions.

Second, Profile 1 (low death anxiety–low future time perspective) represents individuals for whom the mediation pathway appears absent or reversed. These individuals demonstrate low levels of both predictor variables and the lowest nutrition awareness. The mediation model would predict that their low death anxiety should be associated with low nutrition awareness via low future time perspective—and indeed this prediction is borne out. However, the variable-centered model cannot distinguish whether these individuals represent the low end of a continuous distribution or a qualitatively distinct subgroup characterized by existential disengagement. The LPA suggests the latter interpretation may be more appropriate.

Third, Profile 2 (moderate death anxiety–moderate future time perspective) represents the statistical majority and likely drives much of the average effect observed in the mediation model. These individuals demonstrate intermediate levels across all variables, consistent with the mediation model’s prediction that moderate predictor levels should be associated with moderate outcomes. However, this profile’s intermediate position may reflect genuine psychological moderation or may simply represent heterogeneity that the three-profile solution could not further differentiate.

The key theoretical insight is that the average mediation effect does not describe a universal process but rather reflects a mixture of distinct subgroups with different underlying dynamics. The positive indirect effect appears to be driven substantially by Profile 3 individuals, while Profile 1 individuals may demonstrate a different (or absent) relationship between death anxiety and health awareness. This heterogeneity has important implications for both theory and intervention, which we develop in subsequent sections.

#### 4.3.3 Mechanistic divergence: why profiles may differ from the average model

The discrepancy between variable-centered and person-centered findings raises a crucial question: why might the mediation pathway operate strongly for some individuals (Profile 3) but not others (Profile 1)?

We propose that the profiles may differ not merely in degree but in the qualitative nature of their psychological processes. For Profile 3 individuals, death anxiety and future time perspective may be functionally integrated—mortality awareness may actively enhance the perceived value of future time, and expansive future perspective may provide a meaningful framework for channeling existential concerns into constructive action. This integration may reflect what we have termed “constructive anxiety,” wherein the combination of existential awareness and temporal openness creates a motivational synergy that neither variable would produce alone.

For Profile 1 individuals, by contrast, low death anxiety and low future time perspective may reflect a fundamentally different psychological state—one characterized not by successful anxiety management but by existential disengagement or avoidance. The absence of death anxiety in this profile may not indicate resolved or well-managed existential concerns but rather a defensive detachment from mortality-related cognition. Similarly, limited future time perspective may not simply represent an accurate perception of temporal constraints but may reflect motivational withdrawal from future-oriented goals. In this state, the mediation pathway cannot operate because its prerequisite conditions—active engagement with mortality and meaningful future orientation—are absent.

This interpretation suggests that the mediation model identifies a process that is conditional on a particular psychological stance toward existence and time. When individuals are actively engaged with both mortality concerns and future possibilities (Profile 3), the pathway from death anxiety through temporal perspective to health awareness operates robustly. When individuals are disengaged from these concerns (Profile 1), the pathway is attenuated or absent. The mediation model’s average effect represents a weighted combination of these distinct processes, obscuring the qualitative differences between them.

### 4.4 Psychological mechanisms underlying profile-specific nutrition awareness

#### 4.4.1 Profile 1—Existential disengagement: mechanisms of reduced health awareness

Profile 1 (low death anxiety–low future time perspective) demonstrated the lowest nutrition awareness among all subgroups. We propose several interconnected psychological mechanisms that may explain this pattern.

Mechanism 1: Avoidant Coping and Mortality Denial. The combination of low death anxiety and low future time perspective may reflect an avoidant coping style characterized by systematic suppression or denial of mortality-related cognition. While such avoidance may provide short-term psychological comfort, it may also reduce the motivational impetus that mortality awareness can provide for health-protective behaviors. Research on death thought accessibility suggests that moderate levels of mortality awareness can enhance self-regulation and health motivation (64), whereas complete avoidance may eliminate this motivational benefit. These individuals may lack the existential “signal” that alerts them to the importance of health preservation.

Mechanism 2: Temporal Foreclosure and Goal Abandonment. Low future time perspective in this profile may represent what we term “temporal foreclosure”—a psychological closure of the future as a meaningful domain for planning and investment. Unlike SST’s adaptive prioritization of present-focused emotional goals, temporal foreclosure may reflect a maladaptive abandonment of future-oriented goals altogether. If the future is perceived as neither long nor meaningful, investment in health behaviors that yield primarily future benefits becomes psychologically unrewarding. Nutrition awareness, which is fundamentally about sustaining future health, may seem irrelevant to individuals who have effectively closed off the future as a domain of concern.

Mechanism 3: Depression and Anhedonia. The low death anxiety in this profile may partly reflect the flattened affective experience characteristic of depression, rather than successful anxiety management. Depressive symptomatology is associated with reduced anxiety across domains and with diminished future orientation (65). If Profile 1 membership is associated with elevated depressive symptoms, the low nutrition awareness may reflect broader motivational deficits rather than specific relationships with death anxiety or time perspective. We examine this possibility in our supplementary analysis of profile characteristics.

Mechanism 4: Learned Helplessness and Health Fatalism. For elderly hospitalized patients, repeated health challenges may engender a sense of helplessness regarding health outcomes. If individuals have come to believe that their health trajectory is beyond their control, both death anxiety (which requires belief that death is avoidable) and future time perspective (which requires belief that the future is shapeable) may be attenuated. This fatalistic stance would naturally be associated with reduced nutrition awareness, since dietary behavior would seem inconsequential for uncontrollable health outcomes.

#### 4.4.2 Profile 2—Normative adaptation: mechanisms of moderate health awareness

Profile 2 (moderate death anxiety–moderate future time perspective) demonstrated intermediate nutrition awareness levels. As the largest subgroup (53.4%), this profile likely represents the normative experience of elderly hospitalized patients adapting to health challenges within typical psychological parameters.

Mechanism 1: Balanced Existential Awareness. Moderate death anxiety may reflect an adaptive balance between mortality awareness and anxiety management—what existential psychologists describe as “authentic” engagement with death that neither denies its reality nor becomes overwhelmed by its implications (66). This balanced awareness may provide sufficient motivation for health-protective behaviors without the psychological costs of excessive anxiety or the motivational deficits of complete avoidance.

Mechanism 2: Realistic Temporal Appraisal. Moderate future time perspective may represent an accurate, reality-based assessment of temporal horizons that neither overestimates nor underestimates remaining life expectancy. This realistic appraisal may support moderate investment in health behaviors—enough to maintain functioning and quality of life, but perhaps not the intensive focus characteristic of Profile 3. The intermediate nutrition awareness of this profile may reflect a pragmatic, “satisficing” approach to health that balances multiple life priorities.

Mechanism 3: Heterogeneity Within the Profile. The intermediate position of Profile 2 across all variables raises the possibility that this profile represents residual heterogeneity that the three-profile solution could not differentiate. Some Profile 2 members may be similar to Profile 1 individuals who score slightly higher on key variables; others may resemble Profile 3 individuals who score slightly lower. This internal heterogeneity would produce the intermediate average scores we observe while masking meaningful individual differences within the profile. Future research with larger samples might identify additional profiles that better differentiate this group.

#### 4.4.3 Profile 3—Constructive anxiety: mechanisms of enhanced health awareness

Profile 3 (high death anxiety–high future time perspective) demonstrated the highest nutrition awareness among all subgroups. We propose several synergistic mechanisms that may explain this pattern.

Mechanism 1: Mortality Salience as Motivational Fuel. High death anxiety in this profile may function not as psychological disturbance but as motivational fuel that energizes health-protective action. Terror Management Theory research has shown that mortality salience can enhance behaviors perceived as life-extending or self-preserving (67). For Profile 3 individuals, the co-occurrence of high death anxiety with high future time perspective may create conditions under which this motivational potential is realized: mortality concerns provide the impetus for action, while expansive temporal perspective provides meaningful goals toward which action can be directed.

Mechanism 2: Future Time Perspective as Meaning Framework. High future time perspective may provide the cognitive-interpretive framework that transforms death anxiety from a paralyzing threat into a mobilizing force. When individuals perceive the future as open, extended, and filled with possibilities worth pursuing, death anxiety may be reinterpreted as a reminder of what is at stake rather than a harbinger of inevitable loss. This reframing may shift the phenomenological experience of death anxiety from helpless dread to urgent motivation. Nutrition awareness may be enhanced because dietary behavior represents a concrete, controllable domain in which individuals can act on their mortality-motivated desire to preserve the valued future they perceive.

Mechanism 3: Self-Efficacy and Perceived Control. The combination of high death anxiety and high future time perspective may be associated with elevated health self-efficacy—the belief that one’s actions can meaningfully influence health outcomes. Unlike Profile 1 individuals who may perceive health as uncontrollable, Profile 3 individuals may maintain strong beliefs in their capacity to influence their health trajectory. This self-efficacy may both enable high future time perspective (the future seems shapeable) and channel death anxiety into constructive action (mortality is a challenge to be addressed rather than a fate to be accepted). Enhanced nutrition awareness would be a natural consequence of this agentic orientation.

Mechanism 4: Positive Reappraisal and Benefit Finding. Research on coping with serious illness has identified positive reappraisal and benefit finding as predictors of adaptive outcomes (68). Profile 3 individuals may be characterized by a cognitive style that seeks meaning and benefit even in adversity. Their high death anxiety may reflect genuine distress about mortality, but their high future time perspective may reflect successful positive reappraisal—a recognition that remaining time, while limited, holds value worth preserving. This cognitive style would naturally extend to nutrition awareness as a domain in which individuals can actively pursue life-enhancing goals.

### 4.5 Demographic characteristics of latent profiles

#### 4.5.1 Gender distribution patterns

Gender distribution differed significantly across profiles (χ^2^ = 107.686, *p* < 0.001). Profile 1 (low death anxiety–low future time perspective) was predominantly female (76.8%), whereas Profile 3 (high death anxiety–high future time perspective) was predominantly male (86.3%). Profile 2 (moderate death anxiety–moderate future time perspective) showed a more balanced but still male-majority distribution (63.5% male).

This striking gender disparity suggests that men and women may adopt fundamentally different existential-temporal adaptation modes in the context of serious illness. The predominance of women in the disengagement profile (Profile 1) may reflect gender-specific coping patterns: research has documented that women are more likely to employ emotion-focused and avoidant coping strategies in response to health threats (69). The high representation of women in Profile 1 may thus reflect a tendency toward emotional disengagement from mortality-related cognition as a protective strategy.

Conversely, the predominance of men in the constructive anxiety profile (Profile 3) may reflect gender differences in problem-focused coping orientations. Men may be more likely to channel death anxiety into action-oriented responses, including enhanced attention to health behaviors such as nutrition. This interpretation aligns with research suggesting that men tend to respond to health threats with active problem-solving approaches (70). The combination of high death anxiety and high future time perspective in Profile 3 may represent a characteristically masculine pattern of confronting rather than avoiding existential concerns.

These gender patterns have important implications for intervention design. Health education programs targeting nutrition awareness may need to be tailored to gender-specific existential adaptation styles, with different approaches for women who may benefit from strategies addressing emotional disengagement versus men who may respond better to strategies that channel existing mortality awareness into health action.

#### 4.5.2 Age patterns: a non-linear relationship

Age differed significantly across profiles (*F* = 6.457, *p* = 0.002), but the pattern was notably non-linear. Both Profile 1 (M = 76.18, SD = 7.15) and Profile 3 (M = 76.56, SD = 6.85) comprised older individuals compared to Profile 2 (M = 72.30, SD = 6.85).

This curvilinear pattern challenges simple Socioemotional Selectivity Theory predictions that older age uniformly predicts reduced future time perspective. Instead, our findings suggest that advanced age may be associated with polarized existential-temporal configurations: some older individuals (Profile 1) demonstrate the expected pattern of reduced death anxiety and contracted future perspective, while others (Profile 3) demonstrate heightened existential awareness combined with maintained or expanded future orientation.

This polarization may reflect divergent developmental trajectories in late life. Some individuals may respond to advancing age with progressive disengagement from existential concerns—a pattern consistent with traditional gerotranscendence theories (71). Others may respond with intensified existential engagement—a pattern consistent with research on “aging well” and maintained psychological vitality in late life (72). The younger average age of Profile 2 suggests that the normative accommodation mode may be more characteristic of the “young-old” (early 70s), while the more extreme configurations (disengagement or integration) may emerge in the “old-old” (mid-to-late 70s) as individuals are forced to confront mortality more directly.

#### 4.5.3 Educational background: complex patterns

Educational background showed a significant but complex relationship with profile membership (χ^2^ = 45.995, *p* < 0.001). Notably, both Profile 1 (67.0%) and Profile 3 (61.0%) had high proportions of individuals with primary school education or below, while Profile 2 showed the most balanced educational distribution with the highest proportion of secondary (30.1%) and tertiary education (33.1%).

This pattern challenges our initial hypothesis that higher education would facilitate constructive anxiety through enhanced cognitive resources for meaning-making. Instead, the data suggest that the normative accommodation profile (Profile 2) is most strongly associated with higher educational attainment, while both extreme profiles (disengagement and integration) are characterized by lower education.

We propose several interpretations of this unexpected finding. First, higher education may promote moderate, balanced engagement with existential concerns—neither the complete disengagement of Profile 1 nor the intense engagement of Profile 3, but rather a pragmatic middle-ground orientation. Educated individuals may have cognitive frameworks that allow them to acknowledge mortality without being overwhelmed, while maintaining realistic rather than either contracted or expanded future perspectives.

Second, the high proportion of lower-educated individuals in both extreme profiles may reflect that less educated populations are more susceptible to polarized existential responses. Without the cognitive buffering that education may provide, individuals may be more likely to either completely disengage from mortality concerns (Profile 1) or to experience intense existential awareness (Profile 3). This interpretation is consistent with research suggesting that education provides psychological resources for emotional regulation and cognitive flexibility (73).

Third, for Profile 3 specifically, the high proportion of lower-educated individuals combined with high nutrition awareness suggests that constructive anxiety may operate through experiential rather than cognitive-analytical pathways. These individuals may channel death anxiety into health awareness through concrete, practical orientations toward self-care rather than through abstract reflection on health and mortality.

#### 4.5.4 Place of residence: urban–rural differences

Place of residence differed significantly across profiles (χ^2^ = 14.335, *p* < 0.001). Profile 1 showed the highest proportion of urban residents (66.1%), followed by Profile 3 (52.1%) and Profile 2 (45.2%).

The concentration of urban residents in the disengagement profile (Profile 1) is somewhat counterintuitive, as urban environments typically provide greater access to health resources and information. However, this pattern may reflect the social isolation and weakened family connections that can characterize urban elderly populations in China (74). Urban elderly may lack the intergenerational family support structures that remain stronger in rural communities, potentially contributing to the existential disengagement and reduced future orientation characteristic of Profile 1.

Alternatively, urban elderly may face different stressors—including faster pace of life, environmental pollution, and reduced social cohesion—that deplete psychological resources for active existential engagement. The rural majority in Profile 2 may reflect the sustained social integration and traditional value systems that support normative psychological adaptation in rural communities.

#### 4.5.5 Marital status: the role of relational context

Marital status showed the most striking differentiation across profiles (χ^2^ = 122.552, *p* < 0.001). Profile 1 showed the highest proportion of married individuals (89.3%), with no unmarried or widowed participants. Profile 3 showed the lowest proportion of married individuals (39.7%) but the highest proportion of widowed (41.1%) and divorced (18.5%) individuals. Profile 2 showed a more heterogeneous distribution across all marital categories.

This pattern directly contradicts our initial hypothesis that marital support would facilitate constructive anxiety through relational motivation. Instead, the data reveal that widowhood and divorce are strongly associated with the high death anxiety–high future time perspective profile, while marriage is associated with the low death anxiety–low future time perspective profile.

Spousal Loss as Mortality Catalyst. The high prevalence of widowhood in Profile 3 (41.1%) suggests that the death of a spouse may function as a powerful mortality salience induction that fundamentally alters existential-temporal orientation. Witnessing a partner’s death provides direct, unavoidable confrontation with mortality that cannot be easily suppressed or denied. This experience may elevate death anxiety while simultaneously—perhaps paradoxically—expanding future time perspective as a response. Widowed individuals may feel motivated to “live for two” or to honor their deceased spouse’s memory by maintaining health and pursuing meaningful goals. The combination of heightened mortality awareness and maintained future orientation may reflect a meaning-making response to spousal loss.

Marriage as Existential Buffer. The concentration of married individuals in Profile 1 (89.3%) suggests that marriage may function as an existential buffer that reduces conscious mortality awareness. Married individuals may experience the presence of a living spouse as a form of symbolic immortality or death denial—the ongoing relationship creates a sense of continuity that diminishes the felt urgency of mortality. Additionally, married individuals may delegate health-related concerns to their spouse, reducing their own direct engagement with mortality and health issues. This interpretation is consistent with research on conjugal buffering effects in health psychology (75).

Divorce as Existential Disruption. The elevated proportion of divorced individuals in Profile 3 (18.5% vs. 10.7% in Profile 1) suggests that marital dissolution may also heighten existential awareness. Divorce, like widowhood, represents a significant life disruption that may prompt existential reflection and reassessment of priorities. Divorced individuals may have been forced to confront questions of meaning, mortality, and future direction during and after the divorce process, potentially contributing to the high death anxiety–high future time perspective configuration.

#### 4.5.6 Length of hospital stay: temporal progression of adaptation

Length of hospital stay showed a significant gradient across profiles (χ^2^ = 69.232, *p* < 0.001). Profile 1 had the highest proportion of short-stay patients (7–14 days: 24.1%; 15–30 days: 32.1%), while Profile 3 had the highest proportion of long-stay patients (61 days or more: 45.9%). Profile 2 was concentrated in the middle range (31–60 days: 43.8%).

Early Hospitalization—Disengagement (Profile 1). In the initial weeks of hospitalization, patients may respond to the health crisis with psychological disengagement and avoidance. The shock of hospitalization and uncertainty about outcomes may trigger defensive withdrawal from mortality-related cognition. Low death anxiety and low future time perspective in early-stage patients may reflect acute denial or suppression responses rather than stable personality characteristics. The relatively high nutrition awareness observed even in this profile may reflect compliance with hospital nutrition protocols rather than internalized health motivation.

Middle-Stage Hospitalization—Accommodation (Profile 2). As hospitalization extends into the second and third months, patients may gradually develop more balanced engagement with their situation. The normative accommodation profile may represent a transitional stage in which initial denial gives way to realistic acceptance. Moderate death anxiety and moderate future time perspective may reflect growing acknowledgment of health challenges combined with maintained hope for recovery.

Extended Hospitalization—Integration (Profile 3). For patients with the longest hospital stays, prolonged confrontation with serious illness may eventually catalyze deeper existential engagement. The high death anxiety–high future time perspective profile may represent a mature adaptation to extended illness in which mortality awareness has been integrated into a meaningful framework for remaining life. These patients have had the most time to process their situation, and the constructive anxiety pattern may reflect successful meaning-making that converts ongoing health challenges into motivation for active engagement with health behaviors including nutrition.

### 4.6 Theoretical implications

#### 4.6.1 From defensive to constructive: reconceptualizing death anxiety in TMT

Our findings challenge the predominantly defensive conceptualization of death anxiety responses within Terror Management Theory. Traditional TMT research has emphasized that mortality salience triggers proximal defenses (suppression, distraction) and distal defenses (worldview validation, self-esteem enhancement) aimed at reducing conscious and unconscious death-related thought accessibility (13, 76). This framework implicitly positions death anxiety as a psychological threat requiring neutralization rather than a potential catalyst for adaptive behavior.

Our observation that death anxiety is positively associated with nutrition awareness suggests a theoretical reorientation: death anxiety may not function solely as a destructive force requiring defensive management but may also co-occur with heightened attention to health-preserving behaviors. This finding aligns with emerging perspectives within existential psychology that recognize the potential growth-promoting functions of confronting mortality (77), but extends this work by demonstrating specific associations with concrete health behavioral intentions rather than abstract meaning-making or post-traumatic growth.

We propose that TMT’s explanatory framework can be extended through the concept of “constructive anxiety”—a pattern wherein mortality awareness is associated with proactive health engagement rather than defensive retreat. Unlike traditional TMT defenses that aim to reduce death-thought accessibility, constructive anxiety may represent a pattern where mortality awareness remains cognitively accessible but is associated with life-affirming behavioral orientations. This proposition does not contradict TMT’s core tenets regarding the anxiety-buffering function of self-esteem and worldview; rather, it suggests that health-promoting behaviors may themselves constitute a form of symbolic self-preservation that simultaneously addresses existential concerns and promotes physical well-being.

Critically, our findings suggest that the distinction between destructive and constructive anxiety patterns may be associated with individual differences in future time perspective, a variable not traditionally incorporated into TMT frameworks. This leads to our second theoretical contribution.

#### 4.6.2 Resolving the health behavior paradox in SST

The SST faces a theoretical puzzle when applied to health behaviors: if limited future time perspective leads to prioritization of immediate emotional satisfaction over long-term goals, elderly individuals should theoretically reduce investment in health behaviors that yield primarily future benefits (62). Yet empirical evidence shows that many older adults maintain or increase health-promoting behaviors despite perceiving limited time horizons (78). Our findings help resolve this paradox by suggesting that future time perspective’s relationship with health behaviors may be contingent on its co-occurrence with existential awareness.

Specifically, our mediation analysis revealed that future time perspective demonstrates a significant indirect effect in the relationship between death anxiety and nutrition awareness. This pattern suggests that SST’s predictions regarding future time perspective and goal prioritization may require qualification: the motivational implications of temporal perspective may differ depending on whether individuals are simultaneously experiencing heightened mortality awareness. Among individuals with elevated death anxiety, higher future time perspective was associated with stronger nutrition awareness—a pattern that SST alone would not predict, since the theory does not incorporate existential threat as a relevant variable.

We propose that future time perspective functions not merely as a motivational orientation (as SST suggests) but also as a cognitive-interpretive framework that is associated with how existential awareness relates to behavioral intentions. When future time is perceived as open and meaningful, death anxiety may co-occur with goal-directed health investment; when future time is perceived as limited or meaningless, the same level of anxiety may co-occur with fatalistic disengagement. This proposition extends SST by identifying existential awareness as a boundary condition for the theory’s predictions regarding health behaviors in later life.

#### 4.6.3 Theoretical integration: the constructive anxiety framework

Our most significant theoretical contribution lies in the integration of TMT and SST insights into a coherent framework explaining the positive association between death anxiety and health awareness. We term this the “Constructive Anxiety Framework,” which proposes the following theoretical mechanisms. First, death anxiety serves as an existential signal that heightens attention to survival-relevant concerns, including health status and self-care. This proposition is consistent with evolutionary perspectives on anxiety as a threat-detection system (79) and with TMT’s recognition that mortality awareness activates self-preservation motivations.

Second, future time perspective functions as a cognitive moderator that is associated with whether this heightened attention is channeled toward approach-oriented (health-promoting) or avoidance-oriented (fatalistic) responses. When individuals perceive the future as open and meaningful, death-related attention may be associated with investment in health preservation; when the future is perceived as closed or meaningless, the same attention may be associated with withdrawal from health-promoting activities.

Third, the indirect effect of future time perspective in the death anxiety–nutrition awareness relationship suggests that temporal cognition may serve as a meaning-making mechanism through which existential distress becomes associated with adaptive behavioral orientations. This proposition integrates TMT’s emphasis on meaning and self-worth as anxiety buffers with SST’s focus on temporal perspective as a goal-organizing framework.

The “high death anxiety–high future time perspective” subgroup identified through latent profile analysis provides empirical support for this integrative framework. This subgroup’s elevated nutrition awareness suggests that the combination of heightened mortality awareness and expansive temporal perspective is associated with the strongest health behavioral intentions—a pattern that neither TMT nor SST would predict independently, but which emerges logically from their theoretical integration.

The theoretical innovations of this study are presented in Table 8.

**Table 8:** Summary of research innovations.

Theoretical gap	Our contribution	Novel concept/proposition
TMT’s “defensive bias”: overemphasis on anxiety reduction, neglect of anxiety utilization	Demonstrated positive association between death anxiety and health awareness; proposed that death anxiety may co-occur with proactive health engagement	“Constructive Anxiety”: a pattern wherein mortality awareness is associated with life-affirming health investment rather than defensive withdrawal
SST’s “health behavior paradox”: inconsistent predictions for health behaviors in elderly populations	Identified existential awareness as a potential moderating factor in SST’s predictions; showed that future time perspective’s relationship with health awareness differs by death anxiety level	Future time perspective as a cognitive-interpretive framework modulating death anxiety’s behavioral correlates
Lack of theoretical integration between TMT and SST regarding health behaviors	Proposed integrative framework positioning future time perspective as mediating mechanism between existential threat and health behavioral intentions	“Constructive Anxiety Framework”: integration of TMT’s existential threat focus with SST’s temporal cognition focus
Variable-centered approaches obscure individual heterogeneity	Identified three existential-temporal configurations with distinct health awareness profiles; demonstrated that average effects misrepresent subgroup-specific patterns	“Existential activation” profile: high anxiety + high future time perspective associated with highest health awareness

### 4.7 Practical and clinical implication

#### 4.7.1 Profile-specific intervention framework

Our latent profile analysis revealed that the relationship between death anxiety and health awareness is not uniform across individuals but varies substantially depending on co-occurring future time perspective. This finding challenges one-size-fits-all approaches to psychological intervention in elderly hospitalized patients and supports the development of a profile-specific intervention framework.

Screening and Assessment Protocol. Before implementing interventions, clinical teams should assess patients’ existential-temporal configurations using brief screening measures. Based on our findings, we recommend a two-stage assessment protocol. The first stage involves administering abbreviated versions of the Death Anxiety Scale and Future Time Perspective Scale during the initial nursing assessment, which can be completed in approximately 3–5 min. The second stage uses scoring algorithms to classify patients into probable profile membership, with high scores on both measures suggesting Profile 3 membership, low scores on both suggesting Profile 1 membership, and intermediate scores suggesting Profile 2 membership. This classification should be updated during extended hospitalizations, as our findings regarding length of stay suggest that patients may transition between profiles over time.

#### 4.7.2 Interventions for profile 1: addressing existential disengagement

The “low death anxiety–low future time perspective” subgroup demonstrated the lowest nutrition awareness and was characterized by predominantly female gender (76.8%), urban residence (66.1%), married status (89.3%), and shorter hospitalization durations. This profile appears to represent existential disengagement rather than successful anxiety management, requiring interventions that gently re-engage patients with mortality awareness while simultaneously expanding future orientation.

Gentle Mortality Salience Activation. Unlike traditional approaches that aim to reduce death anxiety, interventions for Profile 1 patients should paradoxically aim to increase healthy engagement with mortality awareness. However, this must be done carefully to avoid triggering defensive withdrawal. We recommend implementing Life Review Therapy with Future Orientation, a structured life review intervention that incorporates explicit discussion of legacy, remaining goals, and desired quality of remaining life. Unlike standard life review that focuses primarily on past achievements, this modified approach should allocate approximately 40% of session time to future-oriented questions such as “What do you still hope to experience?” and “How do you want to be remembered for how you lived your final years?” This approach draws on our finding that mortality awareness, when combined with future orientation, is associated with enhanced health motivation.

Spousal Involvement and Relational Motivation. Our finding that 89.3% of Profile 1 patients are married, compared to only 39.7% of Profile 3 patients, suggests that marital relationships may function as existential buffers that reduce direct mortality engagement. Rather than viewing this as problematic, interventions can leverage spousal relationships to enhance health motivation through alternative pathways. Dyadic Health Goal-Setting involves including spouses in nutrition counseling sessions, framing dietary improvements as shared couple goals rather than individual health responsibilities. This approach maintains the relational buffer while directing it toward health-promoting outcomes. Additionally, Spouse-Mediated Future Time Perspective Enhancement encourages spouses to discuss shared future plans, such as upcoming family events, travel aspirations, and grandchildren’s milestones, thereby expanding patients’ future time horizon through relational rather than individual mechanisms.

Addressing Gender-Specific Patterns. The predominance of women in Profile 1 (76.8%) suggests the need for gender-sensitive intervention approaches. Given research suggesting that women may be more responsive to emotion-focused and relationally-oriented interventions, we recommend Emotion-Focused Coping Enhancement, which helps female Profile 1 patients identify and process emotions related to illness and hospitalization that may be suppressed through existential disengagement. By providing safe spaces for emotional expression, interventions may facilitate re-engagement with existential concerns. Social Connection Enhancement is also recommended, as female patients may respond better to interventions emphasizing social aspects of nutrition, such as shared meals, cooking for family, and food-related traditions, rather than purely health-focused messaging.

#### 4.7.3 Interventions for profile 2: facilitating progression toward constructive anxiety

The “moderate death anxiety–moderate future time perspective” subgroup represents the majority of patients (53.4%) and demonstrated intermediate nutrition awareness. This profile showed the most balanced gender distribution (63.5% male), younger average age (72.3 years), and concentration in mid-range hospitalization durations (31–60 days). Interventions for this group should aim to facilitate progression toward the more adaptive Profile 3 configuration.

Future Time Perspective Enhancement. Given that Profile 2 patients already demonstrate moderate death anxiety, the primary intervention target should be enhancing future time perspective to shift them toward the “constructive anxiety” configuration observed in Profile 3. Possible Selves Intervention is a structured intervention asking patients to articulate detailed visions of their “possible future selves” at 6-month, 1-year, and 2-year timepoints. Based on research showing that vivid future self-visualization enhances future orientation, patients should be guided to describe their envisioned future health status, activities, relationships, and sources of meaning. Nutritional goals should be explicitly linked to these possible selves, such as “I want to be able to cook for my grandchild’s birthday next year, which means I need to maintain my strength through good nutrition.”

#### 4.7.4 Interventions for profile 3: sustaining and channeling constructive anxiety

The “high death anxiety–high future time perspective” subgroup demonstrated the highest nutrition awareness and represents the “constructive anxiety” configuration that our theoretical framework identifies as adaptive. This profile was predominantly male (86.3%), characterized by high rates of widowhood (41.1%) and divorce (18.5%), longer hospitalization durations (45.9% at 61 + days), and older age (76.6 years). Interventions for this group should focus on sustaining the adaptive configuration, preventing anxiety from becoming overwhelming, and channeling existential motivation into comprehensive health behaviors.

Anxiety Channeling and Optimization. Health Behavior Action Planning capitalizes on Profile 3 patients’ elevated mortality awareness and future orientation by translating their existential motivation into specific, actionable health behavior plans. Unlike general health education, this intervention should explicitly acknowledge and validate patients’ death anxiety while channeling it into concrete nutritional goals. For example: “Your awareness of life’s preciousness is actually a strength. Let us use that awareness to create a specific plan for how you’ll nourish your body to maximize your quality of life.” Mortality-Integrated Health Identity Development helps patients incorporate their heightened mortality awareness into a coherent health identity. Drawing on identity-based motivation theory, interventions should help patients see healthy eating not merely as a behavior but as an expression of who they are—someone who values and actively preserves their remaining life. This approach sustains the constructive anxiety pattern by giving it stable identity foundations.

Preventing Anxiety Escalation. While Profile 3’s high death anxiety is associated with positive health outcomes, there is risk that anxiety could escalate to maladaptive levels. Anxiety Titration Monitoring involves regular assessment of anxiety levels to ensure they remain within the “constructive” range. If anxiety escalates to the point of sleep disruption, panic symptoms, or functional impairment, interventions should temporarily shift toward anxiety management while preserving future time perspective. Existential Resilience Building involves teaching cognitive strategies for maintaining the constructive anxiety configuration under stress. This includes helping patients distinguish between productive mortality awareness that motivates action and unproductive rumination that paralyzes, along with developing cognitive flexibility to shift between engaging with and stepping back from mortality-related thoughts.

#### 4.7.5 Integrated interdisciplinary team structure

The profile-specific intervention framework requires an interdisciplinary team structure that integrates psychological, nutritional, and nursing expertise. Unlike generic recommendations for “interdisciplinary collaboration,” we propose a specific team configuration derived from our findings.

Core Team Composition. The Existential-Temporal Assessment Specialist, a clinical psychologist or advanced practice nurse with training in existential therapy and time perspective assessment, should oversee profile classification and intervention selection. A Profile-Matched Nutrition Counselor should be a registered dietitian trained in psychologically-informed nutrition counseling who can adapt nutritional messaging to patients’ existential-temporal configurations. A Nursing Care Coordinator should be responsible for integrating profile-specific approaches into daily care, monitoring transitions between profiles during extended hospitalizations, and coordinating family involvement. Weekly Interdisciplinary Rounds. Profile-focused case conferences should discuss each patient’s current profile classification, intervention progress, and any evidence of profile transition. These rounds should specifically address which patients are showing signs of moving from Profile 1 toward Profile 2 or 3, which Profile 3 patients may be at risk for anxiety escalation, and how nutritional interventions should be adapted based on psychological assessment.

### 4.8 Cultural context and cross-cultural considerations

#### 4.8.1 Confucian influences on death anxiety and future orientation

Chinese cultural attitudes toward death and the future are profoundly shaped by Confucian philosophical traditions that have influenced Chinese society for over two millennia. Unlike Western philosophical traditions that often conceptualize death as an individual existential confrontation, Confucian thought embeds mortality within a framework of intergenerational continuity, filial obligation, and collective identity (80).

Death as Relational Rather Than Individual. In Confucian tradition, death is understood less as the termination of individual existence and more as a transition within an ongoing chain of ancestral connection. The concept of filial piety extends beyond death, with descendants maintaining relationships with deceased ancestors through ritual practices (81). This cultural framework may fundamentally alter the phenomenological experience of death anxiety in Chinese elderly patients compared to their Western counterparts. Our finding that death anxiety was positively associated with nutrition awareness may be partly explained by this relational conceptualization. For Chinese elderly patients, death anxiety may encompass not only fear of personal annihilation but also concern about fulfilling one’s role in the intergenerational chain—being present for grandchildren’s achievements, maintaining family harmony, and eventually becoming a venerated ancestor rather than a burden. These relationally-embedded concerns may naturally connect mortality awareness to health-preserving behaviors, as maintaining health becomes an expression of filial responsibility toward both ancestors and descendants.

Future Time Perspective as Lineage Continuity. The Confucian emphasis on lineage continuity may shape how Chinese elderly individuals conceptualize future time perspective. Rather than viewing the future primarily in terms of individual goals and achievements, Chinese patients may perceive future time through a lens of family continuity and intergenerational legacy. This cultural framework may explain why future time perspective demonstrated such a strong mediating role in our study: for Chinese elderly patients, an expanded future orientation may represent not merely personal optimism but a sense of continued relevance within the family lineage. The identification of the “high death anxiety–high future time perspective” profile as demonstrating the highest nutrition awareness gains additional meaning within this cultural framework. These individuals may be those who most strongly experience the Confucian tension between mortality awareness and intergenerational obligation—their elevated death anxiety may reflect deep concern about fulfilling familial responsibilities, while their high future time perspective may reflect continued investment in the family’s future beyond their own lifespan.

#### 4.8.2 Collectivism, social embeddedness, and health motivation

Chinese culture is characterized by collectivist orientations that prioritize group harmony, interdependence, and social obligation over individual autonomy and self-expression (82). These collectivist values have profound implications for understanding how death anxiety relates to health behaviors.

Health as Social Responsibility. In collectivist Chinese culture, maintaining one’s health is not merely a matter of individual well-being but carries significant social and familial implications. Poor health burdens family members who are culturally obligated to provide care, disrupts family harmony, and may bring shame upon the family. This cultural framing may partially explain the positive association between death anxiety and nutrition awareness observed in our study: Chinese elderly patients experiencing death anxiety may be motivated to maintain health not primarily to extend their own lives but to minimize burden on family members and fulfill their social roles as long as possible. This interpretation is supported by our finding regarding marital status differences across profiles. The concentration of married individuals in Profile 1 (low death anxiety–low future time perspective) may reflect the collectivist pattern wherein marital relationships provide existential buffering through social embeddedness—married individuals may experience their identity as more collectively distributed, reducing individual mortality salience. Conversely, the high prevalence of widowhood in Profile 3 (high death anxiety–high future time perspective) may reflect how spousal loss disrupts this collective identity buffer, forcing individuals to confront mortality more directly while also potentially intensifying concern about remaining family obligations.

#### 4.8.3 Cultural attitudes toward aging: from veneration to anxiety

Traditional Chinese culture holds elderly individuals in high regard, with Confucian values emphasizing respect for elders and the wisdom that comes with age. However, contemporary China is experiencing rapid social changes that may be altering these traditional attitudes, creating a complex cultural landscape for elderly hospitalized patients (83).

Shifting Intergenerational Dynamics. China’s one-child policy (1979–2015) has created a “4–2-1” family structure in which a single adult child may be responsible for two parents and four grandparents. This demographic shift has transformed the traditional expectation that elderly parents would be cared for by multiple children, potentially intensifying elderly individuals’ concerns about becoming burdensome. Our finding that death anxiety is positively associated with nutrition awareness may reflect this cultural anxiety—maintaining health becomes crucial when family resources for elder care are stretched thin.

Modernization and Death Attitudes. Rapid modernization in China has introduced Western medical frameworks and attitudes that may coexist uncomfortably with traditional Chinese approaches to death and dying. Elderly hospitalized patients may navigate between traditional acceptance of death as a natural transition and modern medical frameworks that treat death as a failure to be prevented. This cultural complexity may contribute to the heterogeneity observed in our latent profile analysis, with different individuals resolving these cultural tensions in different ways.

### 4.9 Limitations and directions for future research

Although this study possesses innovative significance in theory and methodology, several limitations warrant cautious interpretation. First, the cross-sectional design reveals inter-variable correlations and mediation patterns but cannot establish causal inferences. Future research is recommended to adopt longitudinal tracking designs or experimental intervention paradigms to verify the dynamic impact of death anxiety on changes in nutrition awareness over extended time dimensions and to identify the temporal mechanisms of future time perspective in the regulatory process.

Although rigorous inclusion and exclusion criteria were adopted to enhance measurement consistency and internal validity, the sampling strategy relied on convenience sampling from three hospitals within a single metropolitan area. As a result, the sample may not fully reflect the heterogeneity of elderly hospitalized patients across different regions, cultural backgrounds, or medical institutions of varying levels. Differences in healthcare accessibility, nutritional services, and psychosocial environments may influence levels of death anxiety, future time perspective, and nutrition awareness. Future research should consider multi-center, cross-regional, or stratified sampling approaches to improve representativeness and strengthen the external validity of the findings.

All variables in this study were assessed through self-report questionnaires, which introduces potential biases that could affect both the size and direction of the results. Although we implemented procedural safeguards such as ensuring anonymity, collecting data independently, and clearly emphasizing honest responses, various forms of bias may still have influenced our measurements. For example, death anxiety might be underreported due to cultural values that emphasize accepting death calmly, potentially weakening its true associations with other variables. Future time perspective may be inflated by optimistic self-enhancement, since acknowledging limited future time can feel like admitting failure—especially for hospitalized patients motivated to demonstrate resilience. Nutrition awareness is particularly susceptible to social desirability bias, as healthy eating carries strong normative value, and research consistently shows a substantial gap between self-reported and objective dietary assessments. These biases could have inflated observed correlations via shared method variance, though they might also introduce measurement error that weakens true relationships—the net effect is difficult to determine. Relative patterns among latent profiles may partly reflect differences in impression management tendencies, so profile-specific differences could stem not only from substantive psychological distinctions but also from response styles. The hospital setting might have triggered health-related response biases beyond typical levels; despite anonymity guarantees, the presence of graduate nursing students as data collectors may have activated compliance motives. Future research must address these limitations through methodological triangulation. Objective measures should include biochemical indicators of nutritional status (e.g., serum albumin, micronutrient levels, inflammatory markers), dietary intake observations using plate waste methodology, physiological indicators of death anxiety (e.g., skin conductance, heart rate variability, cortisol responses to death salience), implicit measures of death attitudes using reaction time paradigms, and behavioral economic tasks assessing future orientation via delay discounting choices. Methodological designs should separate predictor and outcome measurements in time, employ multi-method multi-trait designs capable of explicitly estimating method variance, and incorporate social desirability scales for statistical control. Mixed methods, including qualitative interviews and behavioral observations, can provide convergent validation less affected by biases specific to quantitative self-report. These enhancements will greatly strengthen the evidentiary foundation of the constructive anxiety framework we propose.

Third, the sample was drawn from geriatric wards in tertiary Grade-A hospitals in eastern China, with group characteristics exhibiting certain regional and medical resource limitations. More fundamentally, the observed relationships among death anxiety, future time perspective, and nutrition awareness are embedded within Chinese cultural frameworks that may shape these constructs in culture-specific ways. Confucian values emphasizing filial piety, intergenerational continuity, and collective identity may fundamentally alter the phenomenological experience of death anxiety compared to Western individualistic contexts. The collectivist orientation of Chinese culture may make health behaviors more readily connected to social obligation and family responsibility, potentially explaining the positive association between death anxiety and nutrition awareness that might not replicate in cultures where health is framed primarily as individual choice. Additionally, Traditional Chinese Medicine frameworks that conceptualize food as medicine and emphasize preventive health cultivation may heighten the salience of nutrition as a domain for expressing health motivation. Future research should extend validation across diverse cultural contexts to examine whether the constructive anxiety pattern is universal or culture-specific, and to identify which elements of the theoretical framework and intervention strategies require cultural adaptation. Cross-cultural comparative studies could specifically test whether collectivist versus individualistic cultural orientations moderate the relationship between death anxiety and health behaviors, and whether the identified latent profiles show similar or different distributions across cultural contexts.

Additionally, while nutrition awareness is an important indicator of health behaviors, it represents only one dimension thereof. Future research could extend the model to other health behavior domains, such as exercise adherence, medication compliance, and sleep management, thereby establishing a more comprehensive death anxiety → time cognition → integrated health behavior model. Simultaneously, it is suggested to combine objective physiological indicators (e.g., inflammatory markers, BMI changes, or nutritional intake records) with psychological variables to construct cross-level models, achieving integrated validation of psychological mechanisms and physical health outcomes.

Finally, future emphasis should be placed on technology-supported intervention directions. With the widespread application of artificial intelligence and wearable devices, specialized “future time perspective assessment and feedback systems” for elderly patients could be developed. Through real-time psychological monitoring and individualized data feedback, anxiety levels and future time orientations can be dynamically evaluated, enabling continuous psychological and nutritional interventions during hospitalization and post-discharge. This will provide innovative pathways for achieving precision nursing and digital psychological health management.

## 5 Conclusion

This study, from dual perspectives of variable-centered and person-centered approaches, systematically examined the associations among death anxiety, future time perspective, and nutrition awareness in elderly hospitalized patients. The findings revealed a significant positive association between death anxiety and nutrition awareness, with future time perspective demonstrating a significant indirect effect consistent with a partial mediation pattern. These results suggest the potential adaptive correlates of death anxiety: in hospital environments, existential distress may co-occur with heightened health behavior awareness, though causal directionality cannot be established from cross-sectional data. Concurrently, latent profile analysis identified heterogeneous subgroups characterized by different patterns of death anxiety and future time perspective, highlighting individual differences in these psychological profiles; the subgroup characterized by high anxiety accompanied by high future perspective demonstrated the strongest nutrition awareness, suggesting that time perspective may be an important factor in understanding the correlates of death anxiety.

This study extends the potential application of terror management theory and socioemotional selectivity theory to clinical geriatric populations, suggesting that death anxiety may not function solely as a negative factor but may be associated with positive health awareness outcomes in certain contexts. The integration of variable-centered and person-centered approaches provides a more comprehensive understanding of both average associations and individual heterogeneity, offering a preliminary framework for understanding the complex relationships among existential concerns and health behaviors in older adults. These findings may inform the development of clinical nursing and psychological interventions, though intervention effectiveness must be established through future experimental research. If the observed associations are confirmed through longitudinal studies, targeted approaches addressing future time perspective could potentially help elderly hospitalized patients leverage existential awareness in service of health management goals.

In summary, this study provides cross-sectional evidence consistent with a potential adaptive function of death anxiety in the health psychology of elderly hospitalized patients and highlights future time perspective as a potentially important associated factor. Future research employing longitudinal designs and experimental interventions is needed to establish causal relationships, examine temporal dynamics, and test the clinical utility of these findings across diverse cultural contexts.

## Data Availability

The raw data supporting the conclusions of this article will be made available by the authors, without undue reservation.
